# 2013 Korean Society of Hypertension guidelines for the management of hypertension: part I–epidemiology and diagnosis of hypertension

**DOI:** 10.1186/s40885-014-0012-3

**Published:** 2015-03-19

**Authors:** Jinho Shin, Jeong Bae Park, Kwang-il Kim, Ju Han Kim, Dong Heon Yang, Wook Bum Pyun, Young Gweon Kim, Gheun-Ho Kim, Shung Chull Chae, The Guideline Committee of the Korean Society of Hypertension

**Affiliations:** Department of Internal Medicine, Hanyang University College of Medicine, Seoul, Korea; Division of Cardiology, Department of Medicine, Cheil General Hospital, Kwandong University College of Medicine, Seoul, Korea; Department of Internal Medicine, School of Medicine, Seoul National University, Bundang, Korea; Department of Internal Medicine, School of Medicine, Chonnam University, GwangJu, Korea; Division of Cardiology, Department of Internal Medicine, Kyungpook National University School of Medicine, Daegu, Korea; Ewha Womans University School of Medicine, Seoul, Korea; Division of Cardiology, Department of Internal Medicine, Dongkuk University College of Medicine, Ilsan, Korea

**Keywords:** Blood pressure, Blood pressure measurement, Cardiovascular complications, Cardiovascular risk, Guidelines, Hypertension, Lifestyle, Organ damage

## Abstract

The standardized techniques of blood pressure measurement in the clinic are emphasized and the indications for ambulatory and/or home blood pressure monitoring are specified more broadly. The epidemiologic findings specific to Korean population related to blood pressure are reviewed. Cardiovascular risk of hypertensive patients are stratified based upon the data of a Korean population cohort study.

## Introduction

Since the publication of the last Korean Hypertension Treatment Guideline in 2004, new studies have been released and new data on antihypertensive drugs introduced. In response to the changes, the Guideline Committee of the Korean Society of Hypertension began to revise its guideline. To perfectly tailor the needs of our clinical practices, the guideline needs to be based on a great deal of studies performed in Korea. Unfortunately, in reality, there is currently a serious shortage of such study results. The Committee therefore decided to establish the guideline in the form of adaptation. The recently released guideline on hypertension from the European Society of Hypertension/the European Society of Cardiology which is designed to encompass the diverse cardiovascular disease risk levels and socioeconomic statuses of the member nations, and which, therefore, has a broad scope of recommendations, was chosen as a model. Accordingly, a large portion of the 2013 Korean guidelines is based on the European recommendations, although some necessary adjustments are made.

## Epidemiology of hypertension

### The classification of blood pressure and hypertension

Hypertension (HTN) is defined as systolic blood pressure (SBP) or diastolic blood pressure (DBP) greater than or equal to 140 or 90 mm Hg, respectively (Table 1). Normal blood pressure (BP) is defined only as both SBP less than 120 mm Hg and DBP less than 80 mm Hg. When SBP is greater than or equal to 120 but below 140 mm Hg and/or DBP is greater than or equal to 80 but below 90 mm Hg, the patient is considered to have prehypertension. Prehypertension is further classified as stage 1 and stage 2 prehypertension. In stage I prehypertension, SBP is greater than or equal to 120 but below 130 mm Hg and/or DBP is greater than or equal to 80 but below 85 mm Hg. In stage 2 prehypertension, SBP is greater than or equal to 130 but below 140 mm Hg and/or DBP is greater than or equal to 85 but below 90 mm Hg. When SBP is greater than or equal to 140 mm Hg and DBP is below 90 mm Hg, the patient is said to have isolated systolic HTN. HTN is further classified as stage 1 and stage 2 HTN. In stage 1 HTN, SBP is below 160 mm Hg and DBP below 100 mm Hg. In stage 2 HTN, SBP/DBP is greater than or equal to 160/100 mm Hg.

**Table 1:** **The classification of blood pressure and hypertension**

**Category**		**Systolic blood pressure (mm Hg)**		**Diastolic blood pressure (mm Hg)**
Normal blood presssure^a^		<120	And	<80
Prehypertension	Stage 1	120–129	Or	80–84
	Stage 2	130–139	Or	85–89
Hypertension	Stage 1	140–159	Or	90–99
	Stage 2	≥160	Or	≥100
Isolated systolic hypertension		≥140	And	<90

^a^Blood pressure with minimal risk for cardiovascular events.

### The risk of high blood pressure

**Figure 1:**
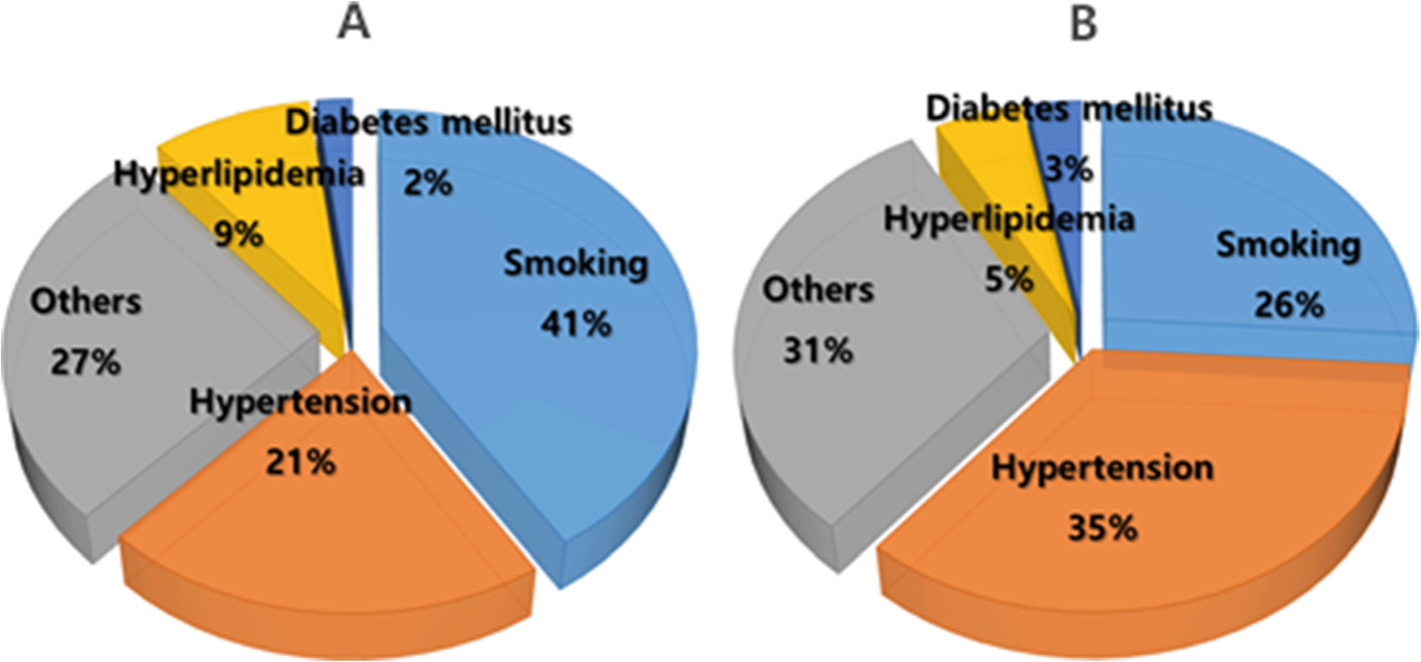
**Attributable risks of hypertension and other cardiovascular risk factors for the coronary artery disease (A) and cerebrovascular diseases (B) in Korean male population (Korean Medical Insurance Corporation study).**

There has been no prospective observational study of a general population to prove the risk of high BP in Koreans. Normal BP has been established as the reference level of BP with the lowest level of cardiovascular (CV) risk in evaluation of the risk of high BP. In the best-documented domestic study of the risk of high BP, which enrolled approximately 100,000 male civil officers and private school teachers (Korean Medical Insurance Corporation [KMIC] study), the hazard ratio for cerebrovascular and coronary artery disease during a 6-year follow-up period was 2.6 for the HTN group relative to the subjects with BP less than 130/85 mm Hg [1,2]. In the nested 248 patient case-control study from the KMIC studies, HTN was the most important risk factor for stroke. Furthermore, the risk of coronary artery disease was 2.51-fold higher in the stage 2 prehypertension group than that in the stage 1 prehypertension group. The group with HTN of greater than or equal to 180/110 mm Hg exhibited a 5.08-fold higher risk than the stage 1 prehypertension group [2,3]. In both Asian and western populations, lifestyle tends to be worse with respect to CV health in subjects with prehypertension than in those with normal BP. In addition, the probability of progressing to HTN and the risk for a CV event were both reported to be higher in the prehypertension group than those in the normal BP group [4-6]. In another paper from the KMIC study, a BP higher than 135/85 mm Hg was associated with the occurrence of hemorrhagic stroke (intracerebral hemorrhage and subarachnoid hemorrhage) in male subjects. As shown in Figure 1, the attributable risks by HTN for cerebrovascular disease and coronary artery disease in men were 35% and 21%, respectively [7]. In addition, for each 20 mm Hg increase in SBP, the relative risks of ischemic stroke, intracerebral hemorrhage, and subarachnoid hemorrhage were 1.79, 2.48, and 1.65, respectively, in men and 1.64, 3.15, and 2.29, respectively, in women [7]. Therefore, the risks of high BP for stroke and coronary artery disease in Korea have been well documented. Moreover, the risk of stroke is more attributable than that of coronary artery disease to HTN.

## The prevalence of hypertension

In the Korean National Health and Nutrition Examination Survey (KNHANES), the age-standardized prevalence of HTN, defined as SBP/DBP of 140/90 mm Hg or higher, was approximately 30% among adults over 30 years of age.

### Changes in the prevalence of hypertension

As shown in Table 2, the prevalence of HTN from the KNHANES was 29.9% and 28.6% in 1998 and 2001, respectively. This figure decreased slightly in 2007 and 2008 and then increased again in 2011. The changes in the prevalence during this period are mainly due to factors other than the true prevalence, such as changes in the survey fields or circumstances [8]. Among adults aged 65 years or older, the prevalence increased between 2007 and 2011 from 49.3% to 58.4% in men and from 61.8% to 68.9% in women. The prevalence of prehypertension in 2001 was 39.8% in men and 30.6% in women. These figures decreased slightly, to 28.4% in men and 18.8% in women, in 2008, similar to the trend in the prevalence of HTN. Overall, less than half of the Korean population has normal BP.

**Table 2:** **Trends in the prevalence of hypertension in the population aged >30 years**

	**1980** ^**a**^	**1990** ^**a**^	**1998** ^**b**^	**2001** ^**b**^	**2005** ^**b**^	**2007** ^**b**^	**2008** ^**b**^	**2011** ^**b**^
All			29.9	28.6	28.0	24.6	26.3	28.5
Men	35.5	33.2	32.5	33.2	31.5	26.9	28.1	32.9
Women	26.9	25.4	26.9	25.4	23.9	21.8	23.9	23.7

Values in % and modified from 2011 Korean National Health Statistics.

^a^The prevalence in 1980 and 1990 were based on the nationwide study for hypertension.

^b^Age adjusted for the estimated population in 2005.

### Age and sex differences in blood pressure

**Figure 2:**
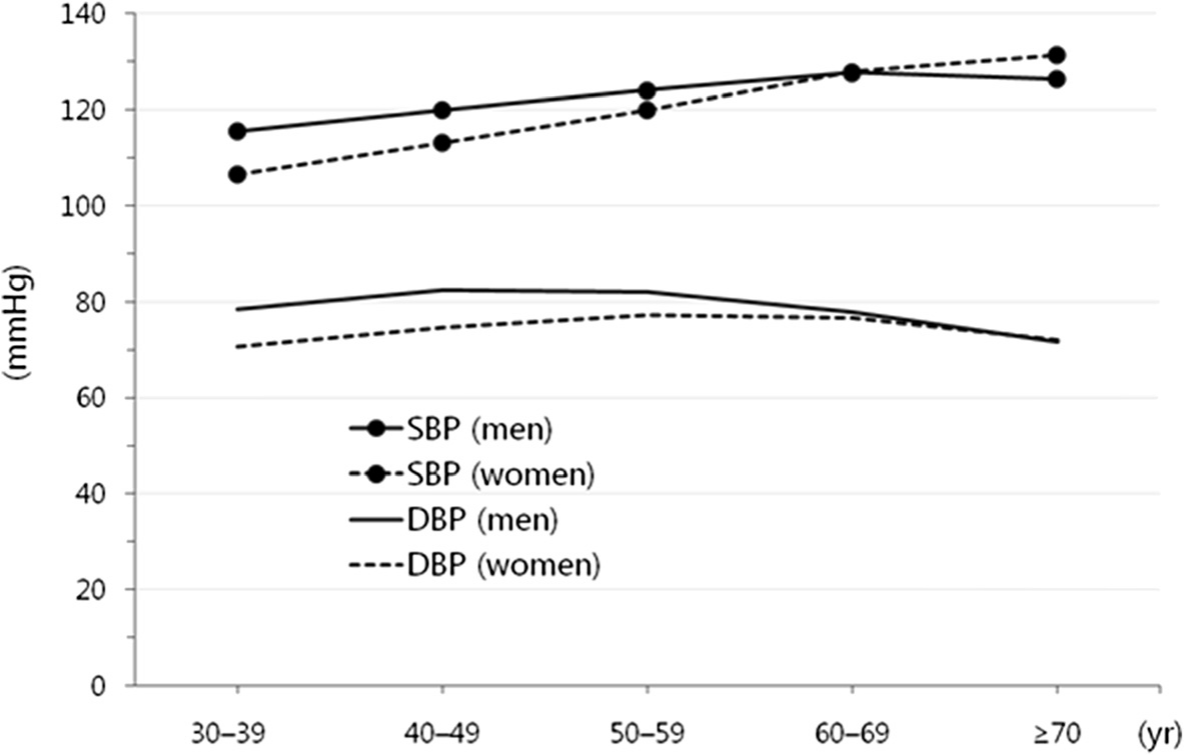
**Levels of blood pressure according to the age groups in male and female Korean populations (2011 Korean National Health and Nutrition Examination Survey data).** *DBP* diastolic blood pressure, *SBP* systolic blood pressure.

BP rises with age, and the difference in BP between the sexes diminishes among those aged more than 60 years. The prevalence of HTN is more than 50% in this older population, i.e., 60 or more years (Figure 2). In general, the prevalence of HTN is 5% to 10% higher in men than in women. Among postmenopausal women, BP increases so rapidly with age that the sex difference becomes trivial within a decade [9].

In KNHANES 2005, the prevalence of HTN among the elderly was 53.7% among those in their 60s and 54.9% among those in their 70s. The prevalence of HTN among those in their 60s was 53.8% in men and 53.6% in women, showing that the sex difference disappeared among those aged 60 or older. As shown in KNHANES 2011, SBP steadily increase with age more than 60 years (Figure 2), whereas DBP decrease, resulting in an increase in the pulse pressure [10].

### The relationship between salt intake and blood pressure

Reducing salt intake is known to decrease BP. Reduction of salt intake is important because the estimated daily salt intake according to KNHANES is approximately 12 g [11]. The report showed that salt intake was higher in the group younger than 40 years than in the other age groups in the cross-sectional analysis of data from 1998, 2001, and 2005. No relationship between salt intake and BP remained after adjustment for energy intake in this analysis [12-14]. However, an independent association between salt intake and BP was reported among people with metabolic syndrome [12-15]. In addition, the urinary sodium/creatinine ratio is more clearly associated with the BP level than with salt intake [16]. Because the salt intake in KNHANES was estimated by using a food questionnaire, which has limited accuracy, and because it is a cross-sectional analysis including both patients with HTN who have not yet changed their diet as well as those who changed their eating habits after diagnosis, additional research is needed. In western countries, reduction of salt intake is reported to lower BP regardless of the relationship between salt intake and BP in the cross-sectional study. There has been no prospective study in Korea to document the BP-lowering effect of a reduction in salt intake.

### Metabolic syndrome and hypertension

In data from the general population in the period from 1998 to 2005, the prevalence of metabolic syndrome, for which the diagnostic criterion for abdominal obesity was a waist circumference of 90 cm in men and 80 cm in women, was reported to be 24.1%, having increased from 22.5% in 1998 to 24.1% in 2001 [17]. High BP is the main component of the metabolic syndrome in men and was observed in 40% of subjects. In women, the low high-density lipoprotein (HDL) cholesterol component was most prevalent (59%), followed by the high BP component observed in 30% of female subjects [18]. Based on the data from KNHANES 2001 and 2005, the prevalence of metabolic syndrome in patients with HTN and prehypertension was 53.3% and 26.2%, respectively, both of which were significantly higher than the 24.1% prevalence in the general population [19]. Metabolic disorder is a determinant of progression from prehypertension to overt HTN [20,21] and thus the key target for lifestyle modification.

## Status of the management of hypertension

In general, it is important to monitor the rates of awareness, treatment, and control of HTN, as these are the key indicators of the quality of HTN management with respect to public health. The HTN awareness rate is defined as ‘the proportion of the subjects who are aware of their physician-diagnosed HTN among all of the subjects with HTN.’ The rate of treatment of HTN is defined as ‘the proportion of the subjects taking antihypertensive drugs at the time of the survey among all of the subjects with HTN.’ The control rate is defined as ‘the proportion of the subjects with BP controlled below 140/90 mm Hg among the subjects taking antihypertensive drugs or among all subjects with HTN.’

The awareness, treatment, and control rates in South Korea are generally improving. According to the data from KNHANES during the period from 2008 to 2011, the awareness rate was 58.5% and 76.1% among men and women aged over 30 years, respectively, which was an improvement relative to the previous data (Table 3). The rate of HTN treatment in 2001 was 22.2% and 37.5% in men and women, respectively, according to the in-depth report of KNHANES 2005. This rate improved to 51.7% and 71.3% among men and women, respectively, in the 2008 to 2011 period (Table 3). The rate of control of HTN in 2001 was quite low at 9.9% and 18.0% among men and women, respectively. However, as of the 2008 to 2011 period, it had increased to 36.9% and 49.4% among men and women, respectively (Table 3). Although there was no clear change in the prevalence of HTN, the mean BP has steadily decreased, especially among patients with HTN.

**Table 3:** **Trends in awareness, treatment, and control rate of hypertension**

**Parameter**	**Year**			
	**1998**	**2001**	**2005**	**2008–2011**
Awareness rate	27.0	36.0	59.8	66.9
Treatment rate	19.1	29.3	47.1	61.1
Control rate for all hypertension patients	7.4	14.9	32.2	42.9
Control rate for treated hypertension patients	22.9	37.0	54.9	69.3

Values in % and standardized for census 2005, Korean National Health Statistics. Criterion for treatment of hypertension: taking antihypertensive drug more than 20 days per month.

The changes in these indicators suggest that the overall management of HTN has improved. However, the rates of awareness, treatment, and control are relatively low among young men in their fourth decade and need further improvement. Despite the low control rate in the young subjects, the control rate among those being treated for HTN did not differ among the age groups. Therefore, early detection and treatment of HTN in younger individuals is very important [8]. According to the 2007 KNHANES data, the proportion of the subjects actively performing three or more of the listed lifestyle modifications (weight control, regular exercise, moderation of alcohol intake, reduction of salt intake, and smoking cessation) was only 38.2% among patients with HTN aged 40 years or older. A campaign or other education to improve lifestyle modification compliance remains warranted. The treatment rate among patients in their 70s is similar to that among those in their 60s, but the control rate among all HTN patients or among treated HTN patients is relatively low among those in their 70s. These findings suggest that more active diagnosis and treatment of HTN in patients aged 70 years or older is needed.

## White coat hypertension and masked hypertension

‘White coat HTN’ is diagnosed when the patient’s BP in the medical office is 140/90 mm Hg or above but the daytime ambulatory or home BP is less than 135/85 mm Hg. If the BP is high both in and out of the office, the patient is considered to have ‘persistent HTN.’ According to the registry data for ambulatory BP monitoring (ABPM) in secondary or tertiary referral centers supported by the Korean Society of Hypertension (KorABP registry) [22], 14.9% of 1,916 subjects who underwent ABPM for the diagnosis of HTN were found to have white coat HTN, as were 25.3% of subjects with HTN diagnosed on the basis of the office BP. In single-center studies from a domestic tertiary hospital, white coat HTN occurred more frequently in women and in men with a low body mass index (BMI) [23]. According to the KorABP registry data, the proportion of subjects in whom daytime ambulatory BP was lower than 135/85 mm Hg and office BP higher than 140/90 mm Hg was 13.5% among all treated subjects and 21.3% among subjects with uncontrolled HTN by office BP [22].

‘Masked HTN’ is defined as the condition in which the office BP is less than 140/90 mm Hg and the daytime ambulatory BP or home BP is 135/85 mm Hg or higher. According to the KorABP registry data, masked HTN was observed in 17.6% of patients who underwent ABPM for the diagnosis of HTN [22]. Masked HTN was also observed in 13.8% of patients taking antihypertensive medication and in 35.1% of subjects with controlled office BP. In a domestic study performed in primary care clinics, the prevalence of masked HTN was 21.2%, and male sex, elderly age, and smoking were the independent predictors of masked HTN [24]. Treatment of HTN in a tertiary care center, number of pills, and a higher fasting blood glucose level have also been associated with masked HTN [25].

There has been no study of white coat HTN and masked HTN in a Korean general population. A study in a western population showed that white coat HTN carries a good prognosis over a follow-up period of 5 years but is associated with increased risk for the onset of HTN and occurrence of CV events over the long-term; therefore, patients with white coat HTN should be monitored regularly [26]. In foreign studies, masked HTN has a similar prognosis as persistent HTN both in the general population and in patients with treated HTN [27,28]. A Korean study reported more severe myocardial damage in patients with masked HTN than in those with white coat HTN [29].

## Diagnosis and clinical evaluation

### Blood pressure measurement

Accurate measurement of BP is necessary for the diagnosis, treatment, and prognostication of individuals with high BP. BP varies according to the environment, body part, and clinical setting of measurement. Therefore, the measurement should be repeated and a standard method should be used.

### Measurement of the office or clinic blood pressure

In an office or clinic, BP is usually measured by the auscultation method using a stethoscope. The subject is seated on a chair with his or her back supported. The cuff is placed on the upper arm and maintained at the level of the heart. A mercury or calibrated aneroid sphygmomanometer is used. An automatic sphygmomanometer that has been validated may also be used. The use of mercury sphygmomanometers is decreasing because of the risk of environmental mercury pollution. The use of an automatic sphygmomanometer is recommended in some countries. After the patient has rested for five or more minutes, the BP is measured by the auscultation method. The measurement is performed two or more times. A cuff with an appropriately sized bladder should be used. The standard bladder for adults is 13 cm wide and 22 to 24 cm long. The use of a bladder with a width of at least 40% of the circumference of the arm and a length of 80% to 100% of the circumference of the arm is recommended.

A cuff of appropriate size as recommended by the manufacturer should be used. A cuff that is too small can produce an artificially high BP reading, whereas a cuff that is too large can produce an artificially low BP reading. If the pulses of the lower extremities are weak, BP is measured in the legs to exclude the possibility of peripheral arterial disease. The upper arm cuff is applied to the ankle and auscultation performed on the dorsalis pedis or posterior tibial artery. A large cuff can be applied to the thigh, using a bladder that is 20% wider than the diameter of the thigh (range, 15 to 18 cm). Auscultation is performed on the posterior tibial artery. Accurate BP measurement is shown in Table 4. Because the measured value varies widely when the pulse is not regular, BP should be measured at least three times [30].

**Table 4:** **Blood pressure measurement using auscultation method**

Standard Procedures of Blood Pressure Measurement
After resting for 5 or more minutes in a quiet, appropriate environment
Avoiding smoking, alcohol, or caffeine before measurement
Measuring 2 or more times at 1- to 2-min intervals in one visit
A cuff with a bladder at least 40% of arm circumference wide; 80% to 10% of arm circumference long (a standard bladder for adults: 13 cm wide; 22 to 24 cm long)
Maintaining the upper arm cuff at the heart level
Inflating the cuff rapidly and deflating slowly at a speed of 2 mm Hg per heart beat
Identifying the blood pressure as the systolic blood pressure at the first Korotkoff sound; the blood pressure as the diastolic blood pressure at the fifth Korotkoff sound
Regarding the blood pressure as the diastolic blood pressure at the fourth Korotkoff sound in pregnancy, arteriovenous shunt, and chronic aortic insufficiency
Taking blood pressure in both arms on the initial visit; subsequently using the arm of higher pressure for measuring blood pressure
Taking blood pressure in legs to exclude peripheral arterial disease, when pulses in the lower extremities are weak
Repeating the measurement three or more times to estimate the average systolic and diastolic pressure in case of arrhythmia
Measuring BP after 1- and 3-min standing in elderly persons and persons with diabetes and suspected orthostatic hypotension

### Home blood pressure measurement

Automated device validated for the measurement of out-of-office BP is more and more popular. Home BP is known to be more accurate to predict CV outcomes and cost-effective in HTN patients [31]. Home BP monitoring becomes important in monitoring of BP during treatment in addition to the diagnosis of HTN. Home BP monitoring is useful to diagnose white coat HTN, masked HTN, and resistant HTN and to titrate the dosage of antihypertensive drugs. Besides, home BP monitoring is known to improve the patient compliance [32]. In general, home BP measured by standardized method can be regarded as a substitute for ambulatory BP. As shown in Table 5, the patient should be educated for the validated device, the time of the day, frequency, and period of measurement in addition to the standardized method of home BP measurement [33]. Multimedia is available at www.koreanhypertension.org. Home BP is lower than clinic BP. HTN can be diagnosed when home BP is 135/85 mm Hg or higher. When making a diagnosis of HTN, it is recommended to measure at least five consecutive days in a week and one to three measurements in each session in the morning and evening. In the morning, it is measured after voiding, within 1 h awakening, and before taking antihypertensive drugs. In the evening, it is measured before sleep. When calculating mean BP, the reading on the first day usually are omitted.

**Table 5:** **Measurement of home blood pressure**

**Requirement**	**Description**
Upper arm cuff	Wrist device is used only when extreme obesity can cause error and the device should be kept at the level of heart
Time of measurement	1. Morning: within 1 h after waking up, after urination, before taking antihypertensive drugs, before breakfast, after 5-min rest in a seated position
	2. Night: before retiring, after 5-min rest in a seated position
	3. Other conditions if necessary
Frequency of measurement	One to three times per occasion
Period of measurement	As long as possible; 1 week or more for the diagnosis of hypertension; over at least 5 to 7 days immediately preceding the visit during follow-up of treatment

### Ambulatory blood pressure measurement

Ambulatory BP measurement provides information on the BP during the daytime, nighttime, and specific periods, for example, early morning. Measurement of BP is performed at 15- to 30-min intervals over a 24-h period. Ambulatory BP measurement provides better prognostic information than does BP measurement in the clinic [34]. The mean daytime ambulatory BP criterion for HTN is the same as for home BP, i.e., 135/85 mm Hg or more (Table 6). Ambulatory BP measurement is useful in clinical conditions such as white coat HTN, masked HTN, resistant HTN, labile HTN, and autonomic dysfunction and also when accurate measurement of BP is required for risk assessment [35]. HTN is regarded as morning HTN when the BP value obtained by home or ambulatory BP measurement in the morning hours is 135/85 mm Hg or more and is higher than those taken before going to bed. Morning HTN is considered to be a risk factor for cardiovascular disease (CVD), particularly stroke [36]. BP shows a diurnal rhythm, being higher during waking hours and lower during sleep. Normally, the average BP is 10% to 20% lower at nighttime than during the day (dipper). A difference of less than 10% (non-dipper) or an increase in the nighttime BP relative to the daytime BP (riser) is associated with increased risk for death, myocardial infarction, and stroke [37]. A decrease of 20% or more (extreme dipper) may be associated with increased risk for ischemic stroke and atherosclerosis [38]. Risers may have autonomic dysfunction and are at increased risk for hemorrhagic stroke [39]. When measuring ambulatory BP, it is important to instruct the patient to engage in his or her ordinary daily activities but to avoid strenuous exercise and also to hold the arm still and in extension during cuff inflation. It is also necessary to educate the patient in how to keep a diary and how to turn off the monitoring device.

**Table 6:** **Criteria for hypertension diagnosis with different methods of measurement**

**Methods**	**Systolic blood pressure (mm Hg)**	**Diastolic blood pressure (mm Hg)**
Clinic or office blood pressure	≥140	≥90
Ambulatory blood pressure		
24 h	≥130	≥80
Day	≥135	≥85
Night	≥120	≥70
Home blood pressure	≥135	≥85

## Evaluation of the patient

Diagnosis and examination are aimed at: 1) differentiating primary and secondary HTN, 2) evaluating the severity of HTN, 3) identifying CV risk factors and lifestyle issues, and 4) searching for CVD, concomitant disease, or subclinical target organ damage that could affect the choice of treatment.

### Symptoms and signs

Patients with HTN most often have no specific symptoms of high BP. High BP is usually found incidentally when hypertensive patients seek health care for other reasons or else is detected in conjunction with symptoms of hypertensive CVD or an underlying secondary cause of HTN. Headache is often considered a symptom of high BP. However, there is no link between high BP and headache except in cases of severe HTN. Headache accompanied by HTN is commonly localized to the back of the head, occurs early in the morning upon awakening, and subsides spontaneously during the day. Some patients with HTN exhibit general, non-specific symptoms of high BP such as dizziness, palpitation, fatigue, and sexual dysfunction. The symptoms of hypertensive CVD are hematuria, blurred vision, dizziness due to transient cerebral ischemia, angina, and shortness of breath due to heart failure. In some rare cases, chest pain due to aortic dissection or aortic aneurysm can occur. Patients with secondary HTN can have specific symptoms and signs suggestive of the underlying cause. For example, patients with sleep apnea syndrome may experience early morning headache, excessive daytime sleepiness, depression, reduced concentration, and nocturnal dyspnea. Patients with primary aldosteronism may have polyuria, polydipsia, and episodes of muscle weakness; those with Cushing’s syndrome, weight gain and emotional instability; and those with pheochromocytoma, episodic headache, palpitation, sweating, and orthostatic hypotension.

### Medical history

The medical history includes: 1) personal history of present illness, past history, and family history; 2) symptoms and signs suggestive of secondary causes of HTN; 3) symptoms and signs of target organ damage; 4) CV risk factors; 5) concomitant diseases; 6) lifestyle factors such as diet, smoking, alcohol consumption, physical activity, exercise, sleep, and personality/psychological state; 7) duration and previous level of high BP, previous treatment and its results, and adverse effects of antihypertensive therapy; 8) use of non-steroidal anti-inflammatory drugs, oral contraceptives, herbs, and other drugs; and 9) socioeconomic status.

### Physical examination

Physical examination of hypertensive patients includes: 1) measurement of BP in both arms at the first visit as well as the pulse rate; 2) measurement of height and weight to calculate the BMI as well as measurement of waist circumference; 3) auscultation for bruits over the carotid arteries, abdomen, and femoral arteries; 4) palpation of the thyroid; 5) examination of the heart and lungs; 6) examination of the abdomen for kidney enlargement, masses, bladder distension, and abnormal aortic pulsation; 7) examination of the lower extremities for edema and palpation of the pulses; and 8) neurological examination. Waist circumference is measured with a tape measure at the level midway between the lowest rib and the iliac crest with the patient in a standing position, at the end of normal expiration, with the abdomen exposed and without compression of the abdominal skin.

### Laboratory examination

Laboratory examination is performed to identify additional CV risk factors, secondary causes of HTN, subclinical organ damage, and concomitant diseases. Routine laboratory tests should be performed before antihypertensive treatment. Other recommended and extended tests may be performed if necessary (Table 7).

**Table 7:** **Laboratory examinations**

**Level**	**Tests**
Routine	12-lead electrocardiogram
	Urinalysis: proteinuria, hematuria, glucosuria
	Hemoglobin, hematocrit
	K+, creatinine, estimated glomerular filtration rate, uric acid, fasting glucose, lipids (total cholesterol, high density lipoprotein cholesterol, low density lipoprotein cholesterol, triglyceride)
	Chest X-ray
	Microalbuminuria: albumin/creatinine (in random urine sample)
Recommended	75 g oral glucose tolerance test or hemoglobin A1c (when fasting glucose ≥ 100 mg/dL)
	Echocardiogram
	Carotid ultrasound: intima-media thickness, plaque
	Ankle-brachial blood pressure index
	Pulse wave velocity
	Fundoscopy (mandatory in diabetes)
	24-h urine protein excretion
	Ambulatory blood pressure/home blood pressure measurements
Extended	Search for asymptomatic organ damage: brain, heart, kidney, vessels
	Search for secondary causes of hypertension

## Cardiovascular risk factors and subclinical organ damage

HTN is typically accompanied by other CV risk factors, so frequently so that reduction of BP alone is insufficient to control the clinical risk for CV events [17]. In some high-risk patients by the estimation of the global CV risk or patients with organ damage, BP-lowering treatment can be initiated even when the BP is below the diagnostic cutoff for HTN. However, there is no risk stratification tool specific for Korean patients with HTN. Table 8 shows the factors in addition to the BP level that are used for evaluation of the risk for future CV events, such as 1) risk factors: age, smoking, obesity, dyslipidemia, increased fasting blood glucose, familial history of premature CVDs, and diabetes mellitus (DM); 2) signs of subclinical organ damage: (micro)albuminuria, left ventricular hypertrophy (LVH), retinopathy, atherosclerosis, and increased arterial stiffness; and 3) clinical CVD: cerebrovascular disease, heart disease, chronic kidney disease (CKD), and peripheral vascular disease [40]. These predictors of the individual patient’s risk are very useful for making clinical decisions, and studies are therefore needed to develop a risk stratification system specific for the Korean population.

**Table 8:** **Cardiovascular risk factors and subclinical organ damages**

**Category**	**Criteria**
Risk factor for cardiovascular disease	Age (men ≥ 45 years old, female ≥ 55 years old) Smoking
	Obesity (body mass index ≥ 25 kg/m^2^) or abdominal obesity (waist circumference male > 90 cm, women > 80 cm) [41]
	Dyslipidemia (total cholesterol ≥ 220 mg/dL, low-density lipoprotein cholesterol ≥ 150 mg/dL, high-density lipoprotein cholesterol < 40 mg/dL, triglycerides ≥ 200 mg/dL)
	Impaired fasting glucose (100 ≤ fasting blood glucose < 126 mg/dL) or impaired glucose tolerance
	A family history of premature cardiovascular disease (men < 55 years old, women < 65 years old)
	Diabetes mellitus (fasting blood glucose ≥ 126 mg/dL, postprandial 2-h glucose (oral glucose tolerance test) ≥ 200 mg/day, or hemoglobin A1C ≥ 6.5%)
Subclinical organ damage and cardiovascular disease	Brain: cerebrovascular accident, transient cerebral ischemia, vascular dementia Heart: left ventricular hypertrophy, angina pectoris, myocardial
	infarction, heart failure
	Kidney: microalbuminuria (range, 30 to 299 mg/day), overt proteinuria (≥300 mg/day), estimated glomerular filtration rate < 60 mL/min/1.73 m^2^, chronic kidney disease
	Blood vessel: atherosclerosis, aortic disease, peripheral vascular disease (ankle-brachial blood pressure index < 0.9), carotid intima-media thickness ≥ 1.0 mm, carotid-femoral pulse wave velocity > 10 m/s
	Retina: stage 3 or 4 hypertensive retinopathy

## Risk stratification system of hypertension

The risk stratification of HTN in South Korea was based on the KMIC data, which were drawn from patients with the following characteristics: 1) registered in the early 1990s, 2) relatively young age range of 35 to 59 years, and 3) relatively high socio-economic status. Therefore, this stratification may have limitations including a tendency to underestimate the absolute risk of HTN and lack of representativeness of the entire population [42]. The lowest CV event risk in the patients with HTN in the KMIC data was 2% to 3% or 2.5% among the patients in their 40s. According to the guidelines presenting risk group by CV event rates [43,44], the average-risk group was those patients with a risk approximately twofold higher than that of the lowest risk group, corresponding to a 10-year CV event rate of 5%. The moderate added risk group was defined as the patients with a risk ≥twofold higher than that of the average risk group, i.e., a 10-year CV event rate of ≥10%. The high added risk group was defined as the group with a risk ≥twofold higher than that of the moderate added risk group, i.e., a 10-year CV event rate of ≥20%. Therefore, the 10-year CV event rates for the lowest, average, low added, moderate added, and high added (including highest added risk group) risk groups were 2.5%, 5%, 5% to 10%, 10% to 15%, and ≥15%, respectively, after consideration of the potential underestimation; these levels correspond to the CV event rates of 2.5%, 5%, 5% to 15%, 15% to 20%, and 20% in the European guidelines [43-45].

In the risk table derived from the KMIC data shown in the Korean Society of Lipidology and Atherosclerosis guideline for the treatment of dyslipidemia 2009 [42], patients with stage 1 HTN who are in their 40s and have no other CV risk factors have a risk of 4.3% to 5.3%; some of them may be at above average risk, whereas the women in this group are at below average risk, i.e., 4.0% to 4.9%. Although the age in the risk table was blocked in 10-year units, preventing observation of any gradual change in risk with respect to age, women with HTN who were in their 50s were at clearly higher than average risk. Therefore, among patients with HTN, men aged 45 years and women aged 55 years were considered to be in the low added risk group. Within the limitation of the imprecise age scale, age greater than those sex-specific cutoff values was regarded as a risk factor. The CV risk was 9.8% to 11% among subjects in their 50s with SBP higher than 130 mm Hg and hypercholesterolemia who were smokers, meaning that even a patient with only stage 2 prehypertension could be stratified as at least moderate added risk if he/she has three risk factors. Subjects in their 60s with stage 2 prehypertension and three risk factors clearly belong to the high added risk group. However, further, better-designed prospective observational studies are needed to provide a more representative or clearer estimation of individual risk. The CV risk can be stratified using the BP level, number of risk factors, evidence of subclinical organ damage, and clinical CVDs, as shown in Table 9.

**Table 9:** **Stratification of global cardiovascular risk in hypertension patients**

**Risk profile**	**Blood pressure (mm Hg)**		
	**Stage 2 prehypertension** **(130–139/85–89)**	**Stage 1 ** **hypertension (140–159/90–99)**	**Stage 2 hypertension (≥160/100)**
No risk factor^a^	Lowest risk group	Low added risk group	Moderate to high added risk group
Risk factor other than DM: 1–2	Low to moderate added risk group	Moderate added risk group	High added risk group
Risk factor ≥ 3 or subclinical organ damage	Moderate to high added risk group	High added risk group	High added risk group
DM, cardiovascular diseases, chronic kidney disease	High added risk group	High added risk group	High added risk group

*DM* diabetes mellitus.

^a^Risk factors: age (men ≥ 45 years old, female ≥ 55 years old), smoking, obesity (or abdominal obesity), dyslipidemia, impaired fasting glucose or impaired glucose tolerance, family history of premature cardiovascular disease, and diabetes mellitus. Ten-year cardiovascular event rates for the lowest, average, low added, moderate added, and high added (including highest added risk group) risk groups were 2.5%, 5%, 5% to 10%, 10% to 15%, and ≥15%, respectively, according to Korean Medical Insurance Company Study data.

## Symptoms of and screening tools for secondary hypertension

Secondary HTN can be diagnosed in approximately 5% of all patients with HTN. Additional testing should be performed when the pretest probability is lower than the sensitivity of the screening tests, as in the following cases: 1) secondary HTN suggested by age, medical history, physical examination, basic laboratory examination, and the severity of HTN; 2) poor response to antihypertensive drugs; 3) BP resistant to previously effective treatment for no apparent reason; and 4) sudden onset of HTN. In some cases, secondary HTN can be cured by surgery or drug therapy. Renovascular HTN is a possibility among patients with HTN beginning at an age of <30 or >55 years, worsening of previously well-controlled HTN, an abdominal bruit, resistant HTN, an increase in the creatinine level of >30% over the baseline level following the administration of an angiotensin converting enzyme (ACE) inhibitor or angiotensin II receptor blocker (ARB), and the presence of atherosclerotic disease in other organs. Screening for renovascular HTN is performed by using the captopril renal scan, Doppler ultrasound, computed tomography, or magnetic resonance angiography. Hyperkalemia with no apparent cause or an incidentally diagnosed adrenal mass are indications for evaluation for hyperaldosteronism. Because paroxysmal and/or refractory HTN accompanied by hyperadrenergic symptoms suggests the possibility of pheochromocytoma, measurement of the catecholamine level in the plasma and/or 24-h urine, CT, magnetic resonance imaging, or radioisotope imaging (I-131 metaiodobenzylguanidine) is indicated (Table 10). In recent years, sleep apnea syndrome has been suggested as the leading cause of secondary HTN, but no treatment strategy for high BP associated with sleep apnea syndrome has yet been established [46].

**Table 10:** **Clinical clues and diagnostic tests of secondary hypertension**

**Diseases**	**Clinical clues**		**Laboratory test**		
	**History**	**Physical diagnosis**	**Chemistry**	**Screening test**	**Additional test**
Parenchymal renal diseases	Urinary tract infection or obstruction, analgesic abuse, familial history of polycystic kidney disease	Abdominal mass (polycystic kidney disease)	Proteinuria, hematuria, pyuria, reduced glomerular filtration rate	Renal US	Further studies for kidney diseases
Renal artery stenosis	Fibromuscular dysplasia, premature hypertension (female), atherosclerotic diseases, sudden onset or worsening of hypertension, resistant hypertension, recurrent pulmonary edema	Abdominal bruit	Rapid worsening of renal function (spontaneous or after ACE inhibitor or ARB treatment)	Kidney size difference >1.5 cm, duplex Doppler US, CT	Magnetic resonance imaging, digital subtraction angiography
Primary aldosteronism	Muscle weakness, premature hypertension, familial history of premature stroke (<40 years old)	Arrhythmia (severe hypokalemia)	Hyperkalemia (spontaneously or after treatment by ACE inhibitor or ARB), incidental adrenal mass	Aldosterone renin ratio (after correction of hypokalemia and disappeared effect of ACE inhibitor or ARB)	Suppression test by saline infusion, fludrocortisone, and/or captopril, adrenal CT, adrenal vein sampling
Pheochromo-cytoma	Paroxysmal hypertension, emergency visit by persistent hypertension with headache, sweat, and/or pallor, familial history	Café-au-lait lesion and neurofibro-matosis neurofibroma	Incidental adrenal mass (extraadrenal mass in some cases)	Metanephrine and/or nor-metanephrine in 24-h urine	Abdominal and/or pelvic CT or magnetic resonance imaging, radioisotope scan using meta-iodobenzyl-guanidine
Cushing syndrome	Rapid weight gain, polyuria, polydipsia, psychiatric problems	Central obesity, mood face, buffalo hump, abdominal striae, hirsutism	Hyperglycemia	Cortisol in 24-h urine	Dexamethasone suppression test

## Abbreviations

ABPM: Ambulatory blood pressure monitoring
ACE: Angiotensin converting enzyme
ARB: Angiotensin receptor blocker
BMI: Body mass index
BP: Blood pressure
CKD: Chronic kidney disease
CV: Cardiovascular
CVD: Cardiovascular diseases
DBP: Diastolic blood pressure
DM: Diabetes mellitus
HDL: High-density lipoprotein
HTN: Hypertension
KMIC: Korean Medical Insurance Corporation
KNHANES: Korean National Health and Nutrition Examination Survey
LVH: Left ventricular hypertrophy
SBP: Systolic blood pressure
